# Melatonin enhances salt tolerance in sorghum by modulating photosynthetic performance, osmoregulation, antioxidant defense, and ion homeostasis

**DOI:** 10.1515/biol-2022-0734

**Published:** 2023-09-27

**Authors:** Mengen Nie, Na Ning, Jing Chen, Yizhong Zhang, Shuangshuang Li, Lue Zheng, Haiping Zhang

**Affiliations:** College of Agronomy, Shanxi Agricultural University, 81 Longcheng Street, Taiyuan, Shanxi, 030000, China; College of Resources Environment and Chemistry, Chuxiong Normal University, 546 Lucheng South Road, Chuxiong, Yunnan, 675000, China; Shanxi Key Laboratory of Sorghum Genetic and Germplasm Innovation, Sorghum Research Institute, Shanxi Agricultural University, 238 Yunhua West Street, Jinzhong, Shanxi, 030600, China; Center for Agricultural Gene Resources Research, Shanxi Agricultural University, 81 Longcheng Street, Taiyuan, Shanxi, 030000, China

**Keywords:** sorghum, salt stress, melatonin, plant physiology, seedling growth

## Abstract

Melatonin is a potent antioxidant that can prevent plant damage caused by adverse stresses. It remains unclear whether exogenous melatonin can mitigate the effects of salt stress on seed germination and seedling growth of sorghum (*Sorghum bicolor* (L.) Moench). The aim of this study was to decipher the protective mechanisms of exogenous melatonin (100 μmol/L) on sorghum seedlings under NaCl-induced salt stress (120 mmol/L). Plant morphological, photosynthetic, and physiological characteristics were analyzed at different timepoints after sowing. Results showed that salt stress inhibited seed germination, seedling growth, and plant biomass accumulation by reducing photosynthetic pigment contents, photosynthetic efficiency, root vigor, and mineral uptake. In contrast, seed priming with melatonin enhanced photosynthetic pigment biosynthesis, photosynthetic efficiency, root vigor, and K^+^ content under salt stress. Melatonin application additionally enhanced the activities of antioxidant enzymes (superoxide dismutase, catalase, ascorbate peroxidase, and glutathione reductase) and increased the levels of non-enzymatic antioxidants (reduced glutathione, ascorbic acid) in the leaves. These changes were accompanied by increase in the leaf contents of soluble sugars, soluble proteins, and proline, as well as decrease in hydrogen peroxide accumulation, malondialdehyde content, and electrolyte leakage. Our findings indicate that exogenous melatonin can alleviate salt stress-induced damage in sorghum seedlings through multifaceted mechanisms, such as improving photosynthetic performance and root vigor, facilitating ion homeostasis and osmoregulation, and promoting antioxidant defense and reactive oxygen species scavenging.

## Graphical abstract

**Figure:**
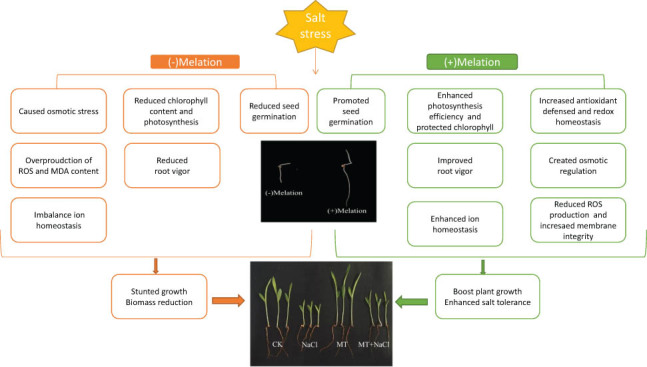

## 1 Introduction

Soil salinity is one of the major abiotic stresses limiting agricultural production worldwide [1], and ∼7% of the world’s land is affected by salt stress [2]. High soil salinity can cause osmotic stress and trigger ion toxicity in plants [3]. It also hinders plant growth and development processes, such as germination, photosynthesis, respiration, ion transport, and nutrient uptake [4], leading to lower yields. Plants have evolved endogenous protective mechanisms to deal with adverse stresses, including activation of antioxidant defense systems and increased accumulation of osmoprotectants. The antioxidant defense system scavenges excessive cellular reactive oxygen species (ROS), and as such, mitigates membrane lipid peroxidation [5]. The accumulation of osmoprotectants, such as proline, soluble sugars, and soluble proteins, neutralizes or mitigates cellular damage from harmful substances by facilitating water uptake [6]. However, these endogenous protective mechanisms are insufficient to overcome the adverse effects of salt stress, which necessitates the application of exogenous protectants.

Melatonin is a potent antioxidant that can mitigate the effects of abiotic stresses on various crops [7–11]. As an indoleamine, melatonin has an outstanding ability to scavenge cellular ROS. Despite its trace amounts in crop plants, melatonin plays a crucial role in physiological regulation and stress response [12]. Like indole acetic acid, melatonin is involved in promoting plant flowering and seed germination, delaying crop senescence [13], and enhancing lateral root development and main root thickening [7]. Exogenous melatonin can improve plant tolerance to abiotic stresses by enhancing endogenous melatonin biosynthesis, antioxidant activity, and osmoprotectant accumulation [5,14]. Elevated melatonin levels also contribute to photosynthesis [9] and ion homeostasis [14] while reducing electrolyte leakage [8]. Melatonin reportedly modulates the expression of genes associated with anthocyanin biosynthesis, transporter protein biosynthesis [15], glucose metabolism, and flavonoid biosynthesis [16].

Sorghum (*Sorghum bicolor* (L.) Moench) in the family Gramineae is a potential crop for production on saline soils because of its wide adaptability and high tolerance to drought, flood, and salinity stresses [17]. Since seed germination and seedling establishment are the most sensitive stages to soil salt concentration, crop plants can be irreversibly damaged if exposed to adverse conditions at these two stages [18]. When soil salinity is higher than 0.3%, the seed germination and emergence of sweet sorghum are affected, and the grain yield is reduced [19]. Therefore, enhancing seed germination and seedling emergence under salt stress is a prerequisite for the promotion of sorghum cultivation and the exploitation of saline soil. An et al. [20] showed that the application of *Bacillus subtilis* strain SN3 could effectively alleviate salt stress-induced damage and improve salt resistance in sorghum seedlings. Whether exogenous melatonin can mitigate the effects of salt stress on seed germination and seedling growth of sorghum remains unknown, despite evidence for its protective role against flooding [21].

In this study, we investigated the protective effects of exogenous melatonin on sorghum seedlings under salt stress from a morphological, photosynthetic, and physiological perspective. The aim of the study was to ascertain the role of melatonin in mitigating salt stress in sorghum and unravel the underlying multifaceted mechanisms. The results could be useful for efficient utilization of saline soils and sustainable sorghum production in saline environments.

## 2 Materials and methods

### 2.1 Materials

Seeds of sorghum variety “Jinza 2002” were provided by the Sorghum Research Institute of Shanxi Agricultural University (Jinzhong, China). Melatonin (chromatographically pure) was purchased from Sigma (USA). Sodium chloride (NaCl, analytically pure) was purchased from Shandong Xiya Chemical Industry Co., Ltd (Linyi, Shandong Province, China).

### 2.2 Methods

#### 2.2.1 Experimental design and setup

Salt stress level (120 mmol/L NaCl) and melatonin solution concentration (100 μmol/L) were selected based on the results of a pre-experiment. Seed germination and seedling growth experiments were conducted in petri dishes and pots, respectively. Each experiment had four treatments and three replications: control (CK; 0 mmol/L NaCl + 0 μmol/L melatonin), MT (0 mmol/L NaCl + 100 μmol/L melatonin), NaCl (120 mmol/L NaCl + 0 μmol/L melatonin), and MT + NaCl (120 mmol/L NaCl + 100 μmol/L melatonin). Each group contained three replicates of 12 plants.

The Petri dish experiment was conducted in a light incubator (16 h/8 h day/night, 25 ± 1°C) on April 15, 2022. Uniform-sized seeds were surface disinfected with 5% NaClO solution for 5 min and then rinsed with distilled water. After that, the seeds were immersed in 0 or 100 μmol/L melatonin solution for 12 h at 25°C in darkness. Then, the seeds were rinsed with distilled water and blotted with filter paper. A total of 21 seeds were placed in each dish (9 cm diameter), followed by the addition of 10 mL of 120 mmol/L NaCl solution or distilled water. The NaCl solution was changed every 24 h to maintain a consistent salt concentration. Seed germination was measured on the third and seventh days.

The pot experiment was conducted in a greenhouse (25/15 ± 2°C day/night) on April 19, 2022. Each pot (12 cm high, 13 cm diameter) was filled with 1 kg of substrate (soil: sand = 2:1, w/w) and ten seeds treated with or without melatonin were sown uniformly at a depth of 4 cm. On the day of sowing, water was applied at 100% field capacity and no stress treatments were applied to all pots. Four and eight days later, 60 mL of 120 mmol/L NaCl solution or distilled water was added to each pot. Twelve days later, 60 mL distilled water was added to each pot. On the 10th, 12th, and 14th days after sowing, seedlings were sampled to measure chlorophyll content, root vigor, antioxidant enzyme activities, and the contents non-enzymatic antioxidants, hydrogen peroxide (H_2_O_2_), malondialdehyde (MDA), and osmoprotectants. Seedling survival, plant height, leaf area (top second leaves), fresh and dry weights, relative water content (RWC), ion contents, and electrolyte leakage (EL) were measured on the 14th day. Leaf photosynthesis was measured on the 25th day.

#### 2.2.2 Germination and growth analysis

Seed germination energy (equation (1)) and rate (equation (2)) were calculated based on the number of germinated seeds.

(1) \[\text{Germination energy}\hspace{.25em}( \% )=\text{The number of germinated seeds on the 3rd day/Total seed number}\times 100,]\]

(2) \[\text{Germination rate}\hspace{.25em}(\text{\%})=\text{The number of germinated seeds on the 7th day/Total seed number}\times 100.]\]

Seedling survival rate (equation (3)) was calculated from the number of surviving seedlings. Seedling height and leaf size were measured using a ruler, with leaf area calculated based on leaf size (equation (4)). The fresh weight (FW) of seedlings was determined using an electric balance. Then, seedlings were deactivated at 105°C and oven-dried at 65°C until constant weight.

(3) \[\text{Seedling survival rate}\hspace{.25em}( \% )=\text{The number of seedlings on the 14th day/Total seed number\times}100,]\]

(4) \[\text{Leaf area}({\text{cm}}^{\text{2}})=\text{Leaf length}\times \text{leaf width}\times \text{0}\text{.75}\text{.}]\]

#### 2.2.3 Seedling physiological measurement

The EL measurement of leaves was done using a conductivity meter [22]. To determine leaf water content, the FW of leaf samples was first measured. Then, the fresh samples were placed in deionized water protected from light overnight. After the saturated fresh weight (TW) was determined, the samples were dried in an oven to constant weight (DW) [23]. RWC was calculated using the following equation:

(5) \[\text{RWC}\hspace{.25em}( \% )=(\text{FW}\mbox{--}\text{DW})/(\text{TW}\mbox{--}\text{DW})\times 100.]\]

The dry samples of crushed leaves (0.1 g each) were extracted with 25.0 mL of 1 mol/L HCl solution. The K^+^ and Na^+^ contents in sample extracts were determined using a flame spectrophotometer [24].

The photosynthetic parameters of the top second leaves were measured using a LI-6400 portable photosynthesizer (LI-COR Inc., USA). The measurements of net photosynthetic rate (Pn), stomatal conductance (Gs), intercellular CO_2_ concentration (Ci), and transpiration rate (Tr) were completed between 10:00 am and 12:00 am on a sunny day. Leaf chlorophyll (a, b) contents were determined by spectrophotometry [25]. Root vigor was analyzed using trimethyl tetrazolium reduction method [25].

Fresh leaf samples (0.5 g) were homogenized in 5 mL of 100 mmol/L phosphate buffer (pH 7.0) using a pre-chilled mortar and pestle. The homogenate was centrifuged at 15,000×*g* for 10 min at 4°C and the supernatant was collected as crude enzyme extract. Superoxide dismutase (SOD) and catalase (CAT) activities were measured using the method described by Li and Zhang [25]. Ascorbate peroxidase (APX) activity was assayed following the method of Nakano and Asada [26]. The method of Foyer and Halliwell [27] was used for quantification of glutathione reductase (GR) activity. SOD, CAT, APX, and GR activities were recorded at wavelengths of 560, 240, 290, and 340 nm, respectively.

Leaf ascorbate (AsA) content was analyzed as described by Jahan et al. [28]. Briefly, a 0.1 g sample of fresh leaves was extracted with 6% perchloric acid and then centrifuged at 4°C at 12,000×*g* for 15 min. The supernatant was added with 200 mM sodium acetate buffer (pH 5.6), and absorbance at the wavelength of 265 nm was recorded before and after incubation with 0.5 units of AsA oxidase for 10 min. The change in absorbance was used to calculate the AsA content. The protocol of Ellman [29] was followed to determine the reduced glutathione (GSH) content. A 0.5 g sample of fresh leaves was homogenized in phosphate buffer (pH 8.0) and then centrifuged at 3,000×*g* for 15 min. The supernatant was incubated with 5,5-dithiobis-2-nitrobenzoic acid for 10 min. The resulting change in absorbance at 412 nm was recorded and used to calculate the GSH content.

Soluble sugar, soluble protein, and proline contents in leaf samples were determined based on the method described by Li and Zhang [25]. To determine H_2_O_2_ content, fresh leaves (0.25 g each) were homogenized on ice with 5 mL of trichloroacetic acid (0.1%) and then centrifuged, and the absorbance of supernatant at 390 nm was recorded [25]. Leaf MDA content was determined using the thiobarbituric acid method [25].

### 2.3 Statistical analysis

The experiments were performed in triplicates (*n* = 3) with the exception of growth parameters (where *n* = 10). SPSS Statistics 18.0 (SPSS Inc., Chicago, IL, USA) was used to conduct one-way analysis of variance. Significant differences between group means were determined by Duncan’s multiple range test (*p* < 0.05). Pearson correlation coefficients were used to evaluate the relationship between seedling morphological and physiological parameters. Principal component analysis was conducted to explore the effects of melatonin on seedling characteristics. Figures were plotted using OriginPro 2021 (OriginLab Corp., Northampton, MA, USA).

## 3 Results

### 3.1 Seed germination and seedling growth

**Figure 1:**
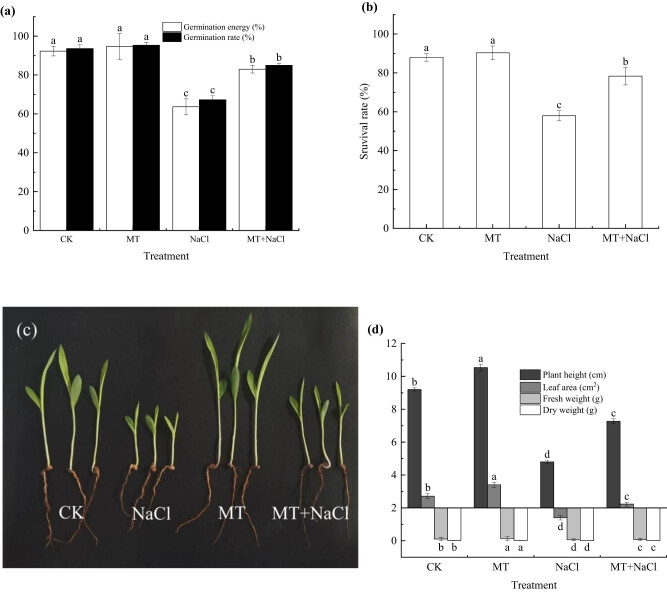
Seed germination (a), seedling survival rate (b), and growth performance (c and d) of sorghum treated with and without melatonin under salt stress. Error bars represent standard deviation of the mean (*n* = 3). Different letters above error bars indicate significant differences among the treatments at the 0.05 level.

The germination energy and rate of sorghum seeds under salt stress were lower than those under non-stress conditions. Both the germination indicators of melatonin-treated seeds considerably increased compared with those of non-treated seeds under salt stress. Specifically, compared with the CK treatment, the seed germination energy and rate of MT treatment increased slightly by 2.47 and 1.75%, respectively; in contrast, those of NaCl treatment decreased significantly by 31.05 and 28.11%, respectively (*p* < 0.05). Despite their remarkable increase in the MT + NaCl treatment relative to the NaCl treatment (30.37 and 26.24%; *p* < 0.05), the seed germination energy and rate were still lower than those of MT and CK treatments (*p* < 0.05). The results of seed germination on the seventh day after sowing mirrored the patterns observed on the third day (Figure 1a).

Compared with the CK treatment, the seedling survival rate of MT treatment showed a minimal increase of 2.58% under non-stress conditions (Figure 1b), whereas a substantial decrease of 34.09% was observed for the NaCl treatment under salt stress on the 14th day after sowing (*p* < 0.05). The seedling survival rate of MT + NaCl treatment was 35.06% higher than that of NaCl treatment (*p* < 0.05), albeit significantly lower than that of CK and MT treatments (*p* < 0.05).

Under non-stress conditions, the seedling plant height, leaf area, fresh weight, and dry weight of MT treatment all increased by 14.49, 25.48, 14.34, and 8.54%, respectively, compared with those of CK treatment (*p* < 0.05). Under salt stress, the four seedling growth indicators of NaCl treatment decreased by 47.83, 48.50, 48.35, and 41.99%, respectively (*p* < 0.05), compared with those of CK treatment. There were remarkable increases in the indicator values of MT + NaCl treatment (21.01, 17.88, 27.43, and 25.20%; *p* < 0.05), compared with those of NaCl treatment (Figure 1c and d).

### 3.2 Seedling water content, electrolyte leakage, and ion homeostasis

**Figure 2:**
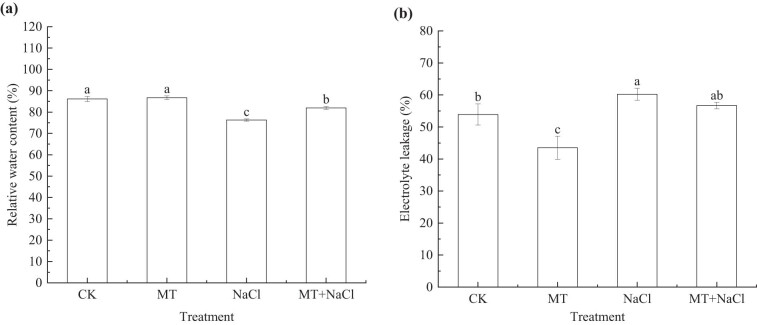
(a) RWC and (b) electrolyte leakage of sorghum seedlings under different treatments. Error bars represent standard deviation of the mean (*n* = 3). Different letters above error bars indicate significant differences among the treatments at the 0.05 level.

On the 14th day after sowing, the RWC of seedlings increased by 6.96% in the MT treatment compared with the CK treatment, and this change was accompanied by a 24.68% decrease in EL (*p* < 0.05). The opposite patterns were observed for the NaCl treatment, with an 11.45% decrease in RWC (*p* < 0.05) and an 11.76% increase in EL (*p* < 0.05). In the MT + NaCl treatment, the RWC was 7.42% higher (*p* < 0.05) and the EL was 5.89% lower than in the NaCl treatment (Figure 2).

On the 15th day after sowing, MT alone caused a 11.36% increase in the K^+^ content (*p* < 0.05) and a 6.96% decrease in the K^+^/Na^+^ ratio (*p* < 0.05) compared with the CK treatment, despite no significant change in the Na^+^ content of seedling leaves. Leaf Na^+^ content significantly increased in response to salt stress, whereas the K^+^ content and K^+^/Na^+^ ratio both decreased in the NaCl treatment compared with the CK treatment. Under salt stress, leaf Na^+^ accumulation was significantly reduced by 28.77% in the MT + NaCl treatment (*p* < 0.05), whereas K^+^ uptake and the K^+^/Na^+^ ratio were increased by 25.67% (*p* < 0.05) and 76.37%, respectively, as compared with the NaCl treatment (Table 1).

**Table 1:** Accumulation of Na^＋^ and K^＋^ in leaves of sorghum seedlings under different treatments. Error bars represent standard deviation of the mean (*n* = 3). Different letters above error bars indicate significant differences among the treatments at the 0.05 level.

Treatment	Na^＋^ (mg g^−1^ DW)	K^＋^ (mg g^−1^ DW)	K^＋^/Na^＋^
CK	2.51 ± 0.32c	14.40 ± 0.20b	5.80 ± 0.72b
MT	2.32 ± 0.26c	16.04 ± 0.54a	6.96 ± 0.54a
NaCl	7.83 ± 0.34a	8.40 ± 0.25d	1.07 ± 0.07c
MT + NaCl	5.58 ± 0.37b	10.51 ± 0.41c	1.88 ± 0.08c

### 3.3 Leaf photosynthetic performance and root vigor

**Figure 3:**
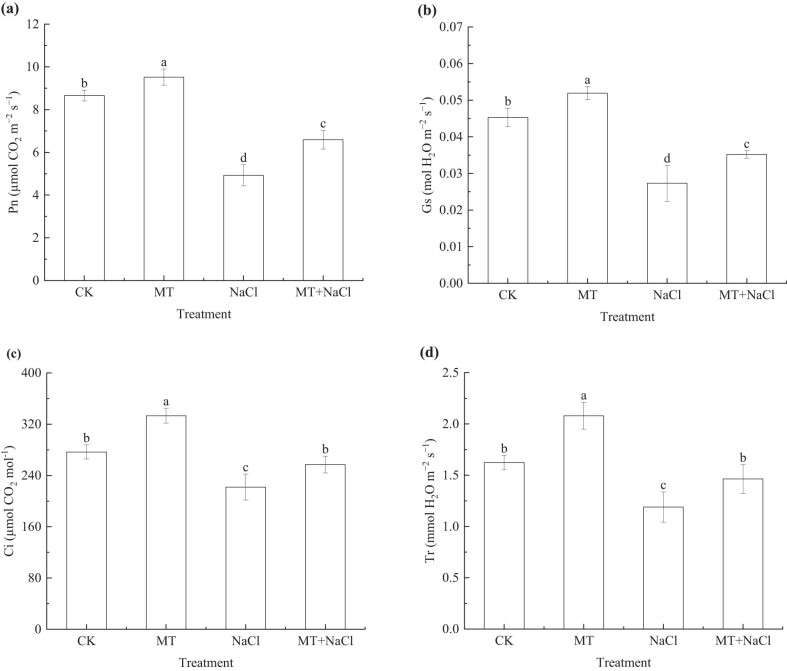
Net photosynthetic rate (Pn; a), stomatal conductance (Gs; b), intercellular CO_2_ concentration (Ci; c), and transpiration rate (Tr; d) of sorghum seedlings under different treatments. Error bars represent standard deviation of the mean (*n* = 3). Different letters above error bars indicate significant differences among the treatments at the 0.05 level.

The photosynthetic parameters of seedling leaves showed consistent patterns in response to salt stress, with lower Pn, Gs, Ci, and Tr values in the NaCl treatment compared with the CK treatment on the 25th day after sowing (*p* < 0.05; Figure 3). NaCl-induced reduction in leaf photosynthetic performance was alleviated in the MT + NaCl treatment. Under salt stress, the Pn, Gs, Ci, and Tr values of MT + NaCl treatment significantly increased by 33.85, 28.94, 15.79, and 22.97% (*p* < 0.05), respectively, compared with the NaCl treatment (*p* < 0.05); however, all parameter values were still lower than in the CK and MT treatments (*p* < 0.05).

**Figure 4:**
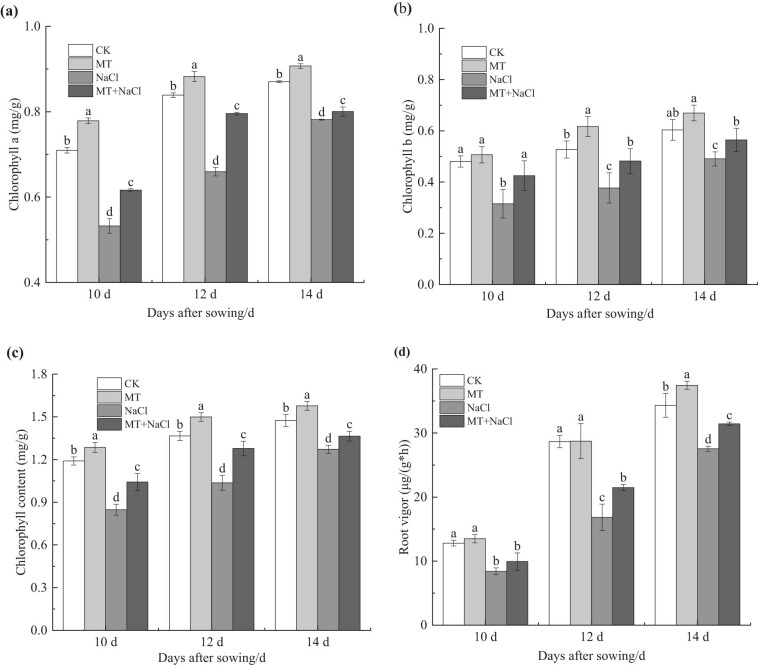
Photosynthetic pigments (a)–(c) and root vigor (d) of sorghum seedlings under different treatments. Error bars represent standard deviation of the mean (*n* = 3). Different letters above error bars indicate significant differences among the treatments at the 0.05 level.

Among different treatments, the photosynthetic pigment contents of seedling leaves were highest in the MT treatment on the 10th, 12th, and 14th days after sowing (Figure 4a–c). Salt stress caused a significant decrease in leaf chlorophyll a, chlorophyll b, and total chlorophyll contents in the NaCl treatment compared with the CK treatment. Although their significant increase in the MT + NaCl treatment compared with the NaCl treatment (a: 15.83–24.10%, b: 15.10–34.82, total: 7.31–23.29%; *p* < 0.05), the pigment contents remained lower than in the CK and MT treatments (*p* < 0.05).

The root vigor of sorghum seedlings increased from the 10th to the 14th day after sowing (Figure 4d). Under non-stress conditions, the root vigor increased by 0.22–9.14% in the MT treatment compared with the CK treatment (*p* < 0.05 on the 14th day). Due to salt stress, the root vigor significantly decreased by 19.76–41.33% in the NaCl treatment compared with the CK treatment (*p* < 0.05). In contrast, seed priming increased root vigor in the MT + NaCl treatment by 14.16–27.72% compared with the NaCl treatment (*p* < 0.05 on the 12th and 14th days).

### 3.4 Leaf antioxidant defense

**Figure 5:**
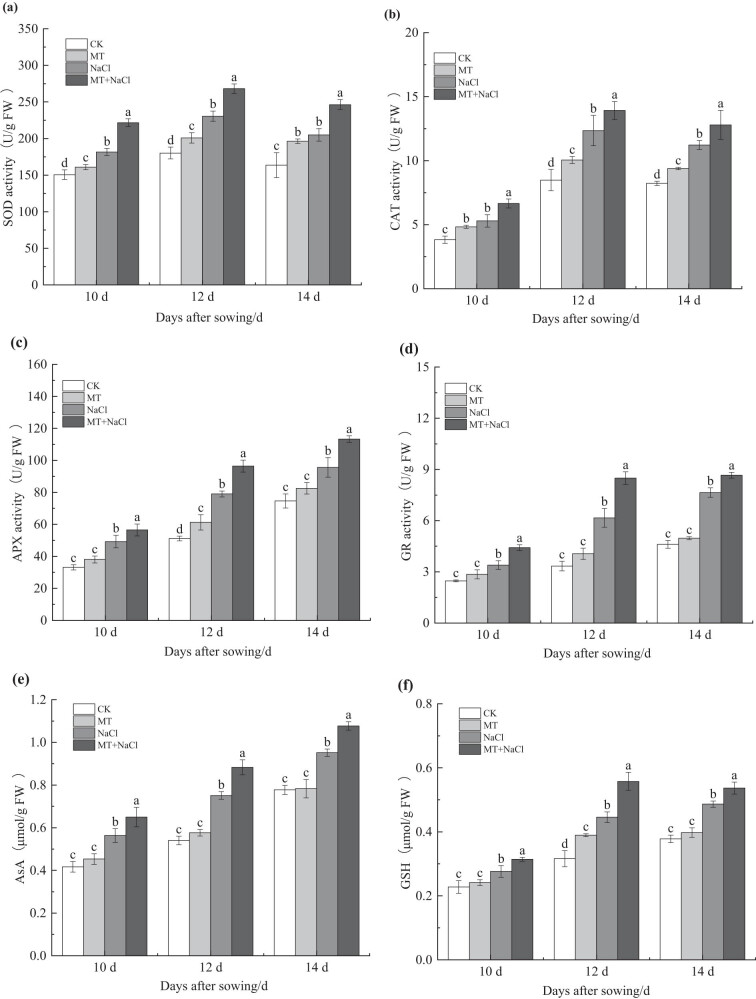
Enzymatic (SOD: a, CAT: b, APX: c, GR: d) and non-enzymatic (AsA: e, GSH: f) antioxidants in leaves of sorghum seedlings under different treatments. Error bars represent standard deviation of the mean (*n* = 3). Different letters above error bars indicate significant differences among the treatments at the 0.05 level.

Under non-stressed conditions, seed priming significantly enhanced the SOD and CAT activities of seedling leaves in the MT treatment compared with the CK treatment, by 6.91–11.55 and 14.13–26.56%, respectively (*p* < 0.05). Leaf APX and GR activities also increased in the MT treatment, albeit not significantly. Under salt stress, all four antioxidant enzymes exhibited markedly higher activities in the leaves of NaCl-treated seedlings compared with the CK treatment (*p* < 0.05). Notably, the SOD, CAT, APX, and GR activities further increased by 13.29–22.01, 13.99–25.63, 14.76–22.01, and 13.32–37.79% (*p* < 0.05), respectively, in the MT + NaCl treatment compared with the NaCl treatment (Figure 5a–d).

The AsA and GSH contents of seedling leaves consistently increased from the 12th to the 14th days after sowing. Compared with the CK treatment, there was no significant difference in leaf AsA and GSH contents in the MT treatment. Nevertheless, both antioxidant contents varied significantly in the NaCl treatment, with the greatest increase of 39.14 and 41.05% on the 12th day, respectively. A further increase in leaf AsA and GSH contents was observed for the MT + NaCl treatment compared with the NaCl treatment, by 13.16–17.57% and 10.29–25.12%, respectively (*p* < 0.05; Figure 5e and f).

### 3.5 Leaf osmoregulation, ROS accumulation, and membrane lipid peroxidation

**Figure 6:**
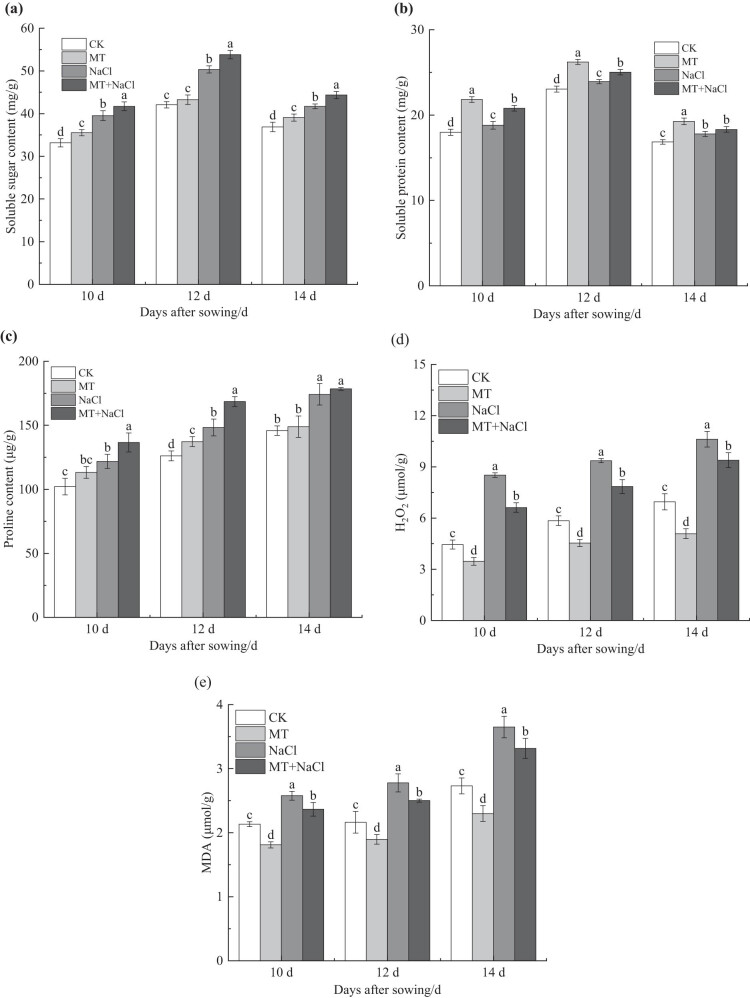
Soluble sugar (a), soluble protein (b), proline (c), H_2_O_2_ (d), and MDA (e) contents in leaves of sorghum seedlings under different treatments. Error bars represent standard deviation of the mean (*n* = 3). Different letters above error bars indicate significant differences among the treatments at the 0.05 level.

The soluble sugar and soluble protein contents of seedling leaves initially increased and then decreased, reaching a maximum on the 12th day (Figure 6a and b). The leaf proline content consistently increased from the 10th to the 14th day (Figure 6c). Salt stress significantly increased the leaf contents of all three osmoprotectants in the NaCl treatment compared with the CK treatment (*p* < 0.05), and seed priming further promoted their leaf accumulation in the MT + NaCl treatment. This promotion effect tended to increase first and then decrease with the extension of treatment time, and was not significant on soluble protein or proline on the 14th day.

Compared with the CK treatment, there was an increase in the H_2_O_2_ and MDA contents of seedling leaves in the NaCl treatment, indicating enhanced ROS production and membrane lipid peroxidation under salt stress. Seed priming led to a substantial decrease in leaf H_2_O_2_ and MDA contents in the MT + NaCl treatment compared with the NaCl treatment (Figure 6d and e).

### 3.6 Correlations between seedling morphological and physiological characteristics

**Figure 7:**
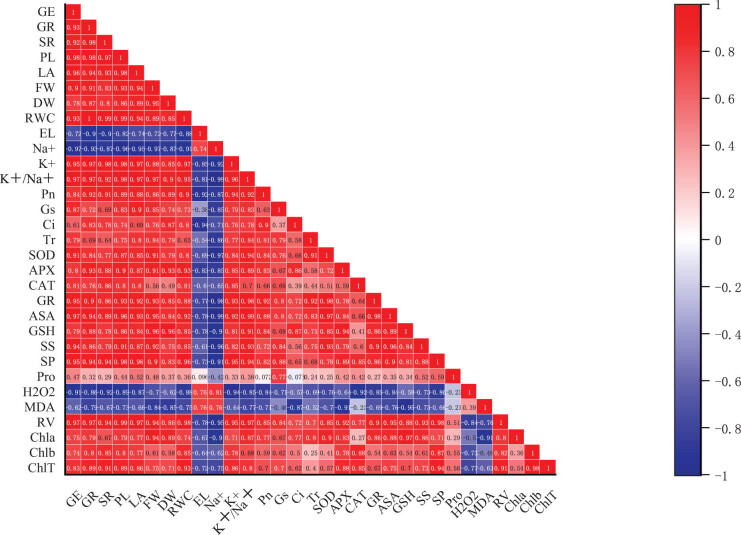
Correlation matrix between leaf morphological and physiological indicators of sorghum seedlings under salt stress. Blue color indicates positive correlations and red color indicates negative correlations. GE: germination energy; GR: germination rate; SR: survival rate, PL: plant height, LA: leaf area, FW: seedling fresh weight, DW: seedling dry weight, RWC: relative water content, EL: electrolyte leakage, Na^+^: Na^+^ content, K^+^: K^+^ content, K^+^/Na^+^: K^+^/Na^+^ ratio, Pn: photosynthetic rate, Gs: stomatal conductance, Ci: intercellular CO_2_ concentration, Tr: transpiration rate, SOD: superoxide dismutase activity, CAT: catalase activity, APX: ascorbate peroxidase activity, GR: glutathione reductase activity, AsA: ascorbate content, GSH: reduced glutathione content SS: soluble sugar content, SP: soluble protein content, Pro: proline content, H_2_O_2_: hydrogen peroxide content, MDA: malonaldehyde content, RV: root vigor, Chla: chlorophyll a; Chlb: chlorophyll b; ChlT: total chlorophyll.

Correlation analysis was conducted on 31 representative indicators to clarify the correlations between the morphological and physiological characteristics of sorghum seedlings under salt stress. Seed germination, seedling survival, and plant growth indicators were all positively correlated with RWC, K^+^ content, K^+^/Na^+^ ratio, photosynthetic parameters (Pn, Gs, Ci, Tr), chlorophyll contents (a, b, total), and root vigor. The germination and growth indicators also had positive correlations with antioxidant enzyme activities (SOD, APX, CAT, GR), non-enzyme antioxidant contents (AsA, GSH), and osmoprotectant contents (soluble sugar, soluble protein, proline). They were negatively correlated with H_2_O_2_ content, MDA content, EL, and Na^+^ content (*p* < 0.05 or 0.01; Figure 7).

Under salt stress, Pn was significantly negatively correlated with EL and positively correlated with RWC, indicating that increased leakage rate of electrolytes was a key factor in the growth of salt-stressed seedlings (Figure 7). Both soluble sugar and protein contents were significantly positively correlated with RWC, AsA, and GSH contents, which means that accumulation of these two osmoprotectants contributed to the regulation of water and non-enzymatic antioxidant contents in seedling leaves.

Both CAT and GR activities were significantly negatively correlated with H_2_O_2_ content (Figure 7), suggesting their role in the reduction of cellular ROS accumulation. K^+^ content and K^+^/Na^+^ ratio were significantly positively correlated with RWC, whereas a significant negative correlation was observed between Na^+^ content and RWC; this indicates that Na^+^ accumulation and K^+^ depletion were key factors affecting leaf water content. Total chlorophyll content and root vigor showed a significant positive correlation with K^+^ content and a significant negative correlation with Na^+^ content, which reflects that Na^+^ accumulation and K^+^ depletion could lead to a decrease in leaf pigment content and root vigor.

### 3.7 Effects of exogenous melatonin on seedling characteristics

**Figure 8:**
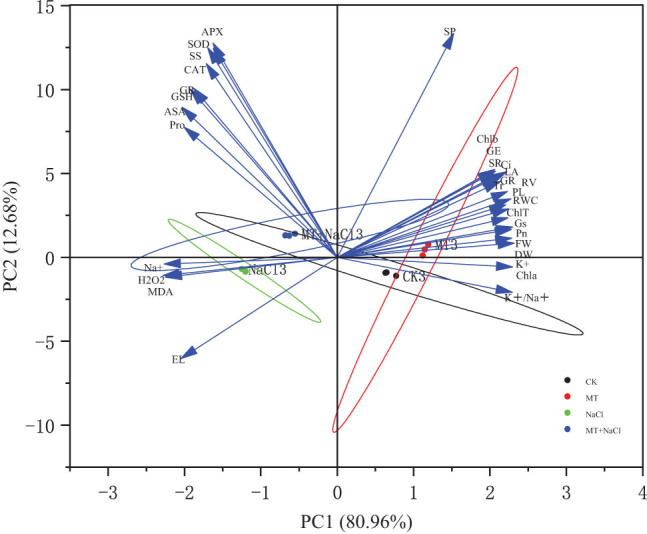
Plots of principal component analysis of sorghum seedling characteristics under different treatments. Abbreviations are defined in Figure 7 caption.

Principal component analysis was conducted to determine the effects of melatonin priming on the morphological, photosynthetic, and physiological characteristics of sorghum seedlings under salt stress. The 31 indicators were grouped into two principal components with contributions of 80.96 and 12.68%, respectively, cumulatively explaining 93.64% of the total variation (Figure 8). Based on the factor loading matrix (Table 2), chlorophyll a content had the highest loading in PC1 and was positively correlated with PC1 scores. Soluble protein content had the highest loading in PC2 and was positively correlated with PC2 scores. Accordingly, the effects of exogenous melatonin on seedling physiological characteristics were mainly attributable to the differences in leaf chlorophyll a and soluble protein contents.

**Table 2:** Factor loading matrix of principal component analysis. Abbreviations are defined in Figure 7 caption.

Indicator	PC1 (80.96%)	PC2 (12.68%)
GE	0.17766	0.13680
GR	0.18290	0.12057
SR	0.18115	0.12912
PL	0.19491	0.09306
LA	0.19029	0.13602
FW	0.19669	0.04834
DW	0.19588	0.03052
RWC	0.18922	0.08328
EL	−0.17538	−0.16093
Na^+^	−0.19421	−0.01056
K^+^	0.19870	0.02260
K^＋^/Na^＋^	0.19657	−0.05554
Pn	0.19552	0.04428
Gs	0.19166	0.06271
Ci	0.17864	0.13044
Tr	0.17684	0.13978
SOD	−0.13907	0.33206
APX	−0.13951	0.34076
CAT	−0.14701	0.30784
GR	−0.17412	0.23865
AsA	−0.16112	0.26870
GSH	−0.16507	0.26748
SS	−0.14548	0.33321
SP	0.13080	0.35618
Pro	−0.17181	0.20695
H_2_O_2_	−0.19555	−0.02887
MDA	−0.19286	−0.03155
RV	0.19104	0.1046
Chla	0.19685	−0.01542
Chlb	0.17600	0.1383
ChlT	0.19230	0.07717

## 4 Discussion

Seed germination and seedling growth are the most sensitive stages of plants to adverse stress [30]. We found that salt stress simulated with 120 mmol/L NaCl considerably inhibited seed germination and seedling survival of sorghum in Petri dishes and pots, respectively (Figure 1a and b). This is compatible with the previous findings in buckwheat [31]. A high concentration of salt can trigger osmotic stress in plant cells, thus causing physiological drought. In the high-salt environment, seeds are unable to take up sufficient water from the external environment for the biosynthesis of enzymes and proteins related to germination. As a consequence, it is difficult to complete cell division and differentiation, as well as embryo growth, which inhibits seed germination [32].

Under NaCl-induced salt stress, seed priming with 100 μmol/L melatonin facilitated seed germination and seedling survival of sorghum. Zhang et al. [33] also found that the application of 300 μmol/L melatonin prominently increased the germination rate of wheat under salt stress. The reason may be that melatonin promotes the formation of globulins and microtubule filaments in seeds, or up-regulates the activities of isocitrate lyase and malate synthase in the glyoxylate cycle, which in turn promotes cell division and elongation to provide energy for seed germination [34,35].

Importantly, the effect of exogenous melatonin is closely related to its concentration applied as well as the crop species treated. For example, foliar spraying of low concentration melatonin (11.61 mg/L) had a remarkable effect on inducing resistance to rust in adzuki bean; this effect diminished with the increase of melatonin concentration [36]. The addition of 0.05 and 1 μmol/L melatonin in Murashige and Skoog medium considerably increased the rooting of rootstock PHL-C, but supplementation with 5 μmol/L of melatonin notably reduced both the number of roots and rooting [37]. When applied at the concentration of 50–200 μmol/L, melatonin promoted seedling growth of tartary buckwheat under AlCl_3_ stress [38]. Therefore, the melatonin concentration for sorghum still needs to be optimized based on stress level and crop variety.

Plant responses to stress are manifested as changes in seedling growth characteristics, which can be used to evaluate plant stress resistance [18]. We found that salt stress inhibited the growth of sorghum seedlings in terms of plant height, leaf area, and fresh and dry weights. Melatonin priming enhanced seedling growth performance under both salt stress and non-stress conditions (Figure 1c and d). Previously, Cui et al. [34] also found that seed priming with melatonin at 1–1,000 μmol/L increased the fresh and dry weights of wheat seedlings under drought stress. In principle, plants accumulate metabolic products through leaf photosynthesis and root nutrient uptake. Salt stress inhibits plant growth primarily by affecting leaf photosynthetic efficiency and root cell activity [39,40]. Additionally, plant growth and biomass formation are challenged by reduced RWC coupled with enhanced ionic and oxidative stresses [4,6].

Chlorophyll, as the most important pigment for photosynthesis in plants, is essential for light energy absorption and conversion. As such, the chlorophyll content not only characterizes plant physiological state, but also provides strong support for photosynthesis [41]. Our results showed that salt stress caused a decrease in chlorophyll a, chlorophyll b, and total chlorophyll contents in leaves of sorghum seedlings (Figure 4a–c), similar to the pattern observed in wheat [42]. Salt stress is likely to reduce chlorophyll pigment biosynthesis by impairing enzyme functions, which in turn triggers structural and functional changes in the protein–pigment–lipid complex [43]. Additionally, a decrease in Fe^2+^ and Mg^2+^ uptake by salt-stress plants is responsible for the reduction of chlorophyll biosynthesis [44].

Seed priming with melatonin increased the chlorophyll a, chlorophyll b, and total chlorophyll contents in seedling leaves. This priming effect was much more pronounced under salt stress than under non-stress conditions. It is most likely that melatonin mitigates salt stress-induced oxidative damage in plants by regulating antioxidant enzyme activities and osmoprotectant contents; this in turn leads to an increase in leaf photosynthetic pigments and a decrease in cell membrane permeability [45]. For example, exogenous application of 200 μmol/L melatonin alleviated the adverse effects of salinity on the chlorophyll content and organic matter biosynthesis in wheat [46]. In the case of bitter melon, melatonin application resulted in increased photosynthetic pigment content and enhanced photosynthesis under salt stress [47].

With respect to photosynthetic performance, NaCl-induced salt stress negatively affected Pn, Gs, Ci, and Tr in sorghum leaves, whereas exogenous melatonin application increased these photosynthetic parameters in the presence/absence of salt stress (Figure 3). The decrease in Pn under salt stress is most likely a result of stomatal limitation, and melatonin could increase the photosynthetic rate by alleviating stomatal limitation, as observed in alfalfa [48]. Accumulating evidence suggests that exogenous melatonin improves plant photosynthesis and salt tolerance by mitigating the inhibitory effects of salt stress on chlorophyll biosynthesis, gas exchange, and photosystem II photochemistry [48,49]. Foliar spraying of melatonin reportedly reduced leaf damage, enhanced photosystem activity, and improved photosynthetic efficiency in tobacco plants under high-Mn stress [8] As for cotton, foliar spraying with 200 μmol/L of melatonin promoted seedling growth and biomass accumulation as a consequence of improved stomatal limitation, alleviated chlorophyll degradation, and enhanced photosynthetic capacity under salt stress [50]. In summary, seed priming with melatonin can enhance the growth and development of crop seedlings by enhancing their photosynthetic performance.

Roots are an important organ for the assimilation and transformation of physiologically active substances during crop growth and development. They are also the first organ to perceive and respond physiologically to adverse stress [51,52]. Therefore, root growth and metabolism directly affect plant shoot development, dry matter accumulation, and final yield formation [51]. Root vigor is a key indicator of root metabolic capacity. Under adverse stress, a more vigorous root system supports more active plant metabolism, as it has a greater capacity for water and nutrient uptake and transport [53]. Our results showed that the root vigor of sorghum seedlings was reduced under salt stress. This reduction was partially attenuated following application of exogenous melatonin (Figure 4d), consistent with previous results obtained from rice [54]. Taken together, seed priming with melatonin can increase seedling root vigor and photosynthetic efficiency under salt stress, it is expected to enhance the translocation of water and minerals from the roots to the shoots and accelerate normal plant metabolism [28], thereby enhancing photosynthetic production and biomass accumulation, but the effects of exogenous melatonin on sorghum yield and quality are still unclear, which warrants further investigations.

When salinity leads to an increase in soil osmotic potential, the plant is unable to absorb sufficient water from the soil, thus triggering osmotic stress [55]. Under salt stress, excessive Na^+^ and Cl^–^ ions enter the plant and accumulate at high levels, causing ion toxicity. Given their similar ionic radius and hydration energy, K^+^ and Na^+^ show a remarkable antagonistic effect. As a result, the presence of excessive Na^+^ often causes K^+^ leakage, which affects plant physiological and metabolic processes [56]. Like wheat [23], sorghum seedlings showed a decrease in RWC in response to salt stress (Figure 2a). Salt stress decreased the K^+^/Na^+^ ratio of sorghum seedlings due to decreased shoot K^+^ content and increased Na^+^ content (Table 1). The K^+^/Na^+^ ratio characterizes the balance of plant ion content and the level of salt stress, so it is often used to evaluate plant salt tolerance [24]. Both salt-induced osmotic stress and ion toxicity can lead to excessive ROS production in plant cells and disruption of membrane stability or integrity, causing leakage of electrolytes and increased lipid peroxidation of cell membranes [50,56]. As we observed in this study, an increase in leaf H_2_O_2_ content (Figure 6d) was accompanied by an increase in EL (Figure 2b) and MDA content (Figure 6e) in sorghum seedlings under salt stress.

Melatonin maintains the osmotic balance of cells by inhibiting Na^+^ uptake and increasing K^+^ levels, as well as alleviating electrolyte leakage and cell membrane lipid peroxidation; these mechanisms mitigate the negative effects of salt stress and improve the overall water status of the plant [43,54]. Our results showed that melatonin seed priming with melatonin increased the K^+^/Na^+^ ratio and RWC while decreasing EL, H_2_O_2_, and MDA accumulation in sorghum seedlings irrespective of salt stress (Figures 2 and 6d and e and Table 1). Similar results have been reported in white beans [5] and chickpea [45]. Melatonin has the ability to modulate Na^+^/K^+^ ion homeostasis in crop plants through upregulating the transcript levels of K^+^ channel genes (*SKOR*), Na/H reverse transporter (*SOS1*), and plasma membrane H-ATPases in root cells [47]. K^+^ reportedly modulates the expression of water channel protein genes and maintains water balance. K^+^ additionally plays an essential role in osmoregulation, stomatal regulation, and ROS detoxification [8]. It was also found that the limitation of Na^+^ influx by melatonin facilitated the accumulation of K^+^, Ca^2+^, and P in bitter melon [47]. The collective results indicate that seed priming with melatonin can promote cell water absorption by regulating ion homeostasis, so as to maintain normal crop growth. Additionally, melatonin application maintains cell membrane stability by reducing cellular ROS accumulation, which is achieved through upregulating the function of the antioxidant system and glyoxalase [45].

Salt stress leads to excessive ROS accumulation in plants, which induces oxidative damage and consequently activates antioxidant defense [5]. In the enzymatic antioxidant system, SOD, CAT, APX, and GR are key enzymes involved in plant responses to stresses. These enzyme activities were all enhanced in leaves of salt-stressed sorghum seedlings in the present study. Seed priming with melatonin further increased leaf SOD, CAT, APX, and GR activities under salt stress (Figure 5a and d). Altaf et al. [57] also reported that 100 μmol/L melatonin application improved antioxidant enzyme activities in tomato seedlings under salt stress (150 mmol/L NaCl), which mirrored the patterns observed in soybean and chickpea [43,45]. Additionally, Luo et al. [58] showed that exogenous melatonin application resulted in higher CAT, SOD, POD, and APX activities in rice seedlings under drought stress by elevating the expression levels of *ALM1*, *OsPOX*, *OsCATC*, and *OsAPX2* genes.

In the non-enzymatic antioxidant system, AsA and GSH are major antioxidants that promote the stability of cell membrane proteins, because they reduce the rate of O_2_^–^ production and catalyze the generation of H_2_O and O_2_ from H_2_O_2_ [43]. While salt stress induced an increase in the AsA and GSH contents of sorghum leaves, melatonin seed priming with melatonin triggered a further increase in these antioxidant contents (Figure 5e and f), which is consistent with the finding by Li et al. [18]. Exogenous melatonin also substantially increased AsA and GSH contents in pepper under combined stress of low temperature and low light [18], as well as in wheat under Cd stress [59]. Furthermore, Luo et al. [60] showed that melatonin treatment enhanced the cold resistance of pumpkin, as plant antioxidant capacity was increased by promoting the AsA–GSH cycle. Collectively, these results indicate that seed priming with melatonin can enhance salt tolerance in sorghum seedlings by activating the antioxidant defense systems, most likely due to the regulation of resistance-related gene expression at the transcriptional level [28].

Myriad studies have shown that organic compounds, such as proline, soluble proteins, and soluble sugars, play major roles in plant osmoregulation and are necessary components for plants to cope with stresses [14,61]. Under the experimental conditions, salt stress resulted in an increase in the soluble sugar, soluble protein, and proline contents of sorghum leaves (Figure 6a–c), in support of previous findings in maize [61]. However, Zhang et al. [33] showed that salt stress induced by 100 mmol/L NaCl decreased the soluble sugar and protein contents while increasing the proline content in wheat. These inconsistent results demonstrate the distinct patterns of osmoprotectants in response to salt stress, depending on crop species. Seed priming with melatonin also increased the soluble sugar, soluble protein, and proline contents in sorghum leaves under salt stress and non-stress conditions (Figure 6a–c). Li et al. [18] observed that melatonin enhanced low-temperature and low-light stress tolerance in pepper by upregulating its soluble sugar and soluble protein accumulation to maintain cellular homeostasis. Additionally, 100 μmol/L melatonin increased the soluble sugar, soluble protein, and proline contents in rice under 80 mmol/L NaCl stress [54].

In summary, exogenous melatonin enhances organic matter accumulation and consequently attenuates NaCl-induced osmotic damage in crop plants, possibly through modulation of gene expression levels. For example, foliar spraying of melatonin was found to increase the leaf proline, fructose, and sucrose contents of rice seedlings under drought stress by upregulating the expression levels of *OsP5CS*, *OsSUS7*, and *OsSPS1* genes [58]. Melatonin was additionally shown to promote endogenous proline accumulation by upregulating *P5CS* and *OAT* gene expression and enzyme activity, as well as reduce proline catabolism by downregulating *PDH* gene expression and enzyme activity [62]. In addition to modulating osmoregulation, soluble sugars and proteins are essential sources of energy for plant growth and development, providing key nutrients for cell survival [1]. Proline is considered to play a role in scavenging ROS [23]. Therefore, seed priming with melatonin may enhance salt tolerance in sorghum seedlings by upregulating osmoprotectant accumulation.

## 5 Conclusions

**Figure 9:**
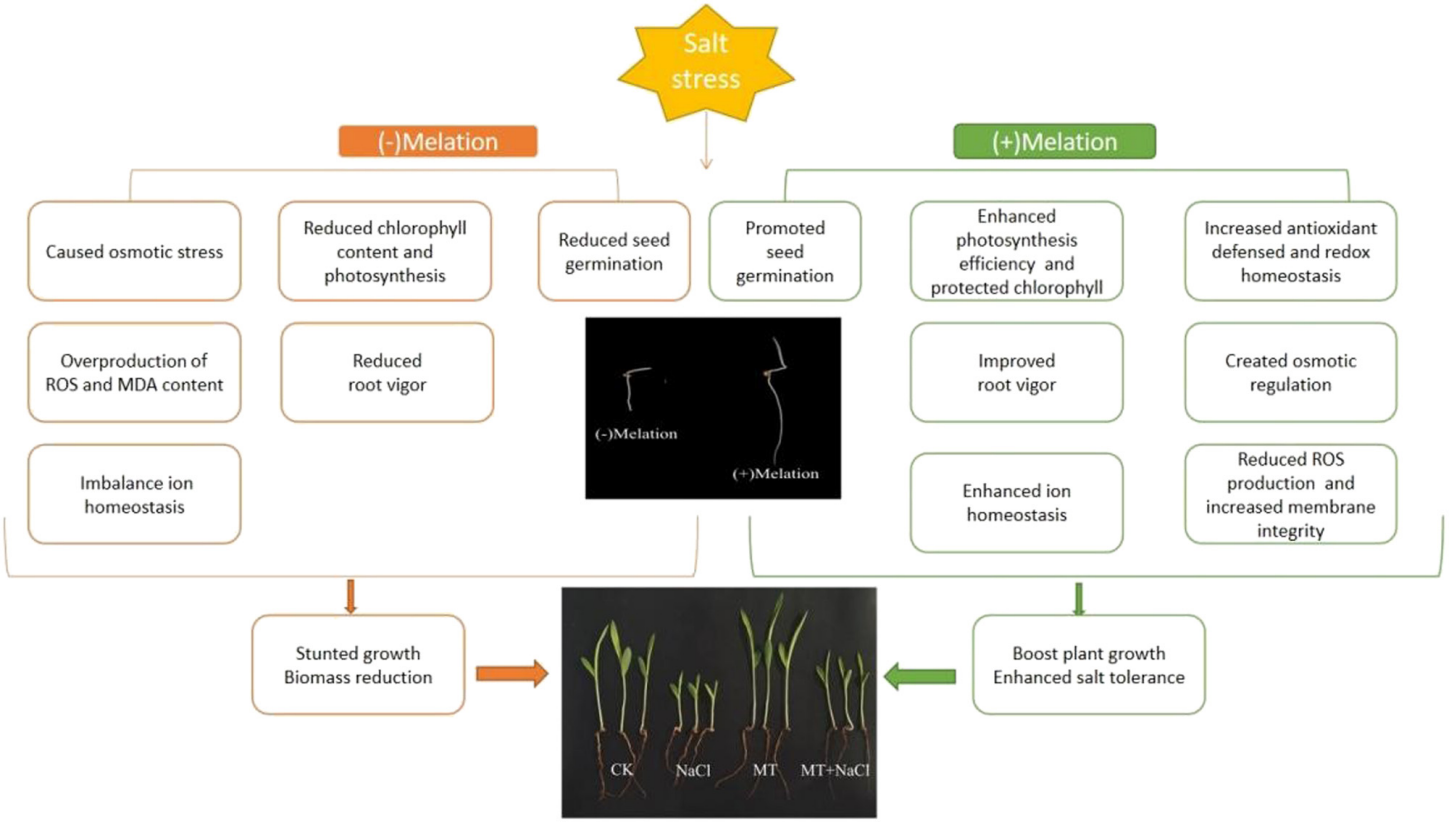
A schematic model figure is showing how melatonin confers salt stress in the sorghum plant.

Salt stress (120 mmol/L NaCl) negatively affected sorghum seed germination and seedling growth of sorghum in petri dishes and pots. Seed priming with melatonin (100 µmol/L) effectively alleviated the effects of salt stress on sorghum, leading to increased seed germination and seedling survival rates (by 26.24 and 35.06%, respectively). Melatonin application improved leaf photosynthetic performance, root vigor, ion homeostasis, antioxidant defense, and osmotic metabolism, in addition to mitigating salt-induced oxidative damage (Figure 9). Chlorophyll a and soluble protein contents were identified as key factors that could explain the protective mechanisms of melatonin on sorghum seedlings. This result of this study can be helpful to improve the stress tolerance and sustainable production of sorghum in saline soils with exogenous melatonin application. Further study of molecular pathways is needed to identify the mechanisms underlying melatonin-mediated enhancement of salt tolerance in sorghum.
